# Glycans from *Fasciola hepatica* Modulate the Host Immune Response and TLR-Induced Maturation of Dendritic Cells

**DOI:** 10.1371/journal.pntd.0004234

**Published:** 2015-12-31

**Authors:** Ernesto Rodríguez, Verónica Noya, Laura Cervi, María Laura Chiribao, Natalie Brossard, Carolina Chiale, Carlos Carmona, Cecilia Giacomini, Teresa Freire

**Affiliations:** 1 Laboratory of Immunomodulation and Vaccine Development, Departamento de Inmunobiología, Facultad de Medicina, UdelaR, Montevideo, Uruguay; 2 Departamento de Bioquímica Clínica, Facultad de Ciencias Químicas, Universidad Nacional de Córdoba, CIBICI-CONICET, Córdoba, Argentina; 3 Departamento de Bioquímica, Facultad de Medicina, UdelaR, Montevideo, Uruguay; 4 Unidad de Biología Parasitaria, Departamento de Biología Celular y Molecular, Instituto de Higiene, Facultad de Ciencias, UdelaR, Montevideo, Uruguay; 5 Cátedra de Bioquímica, Departamento de Biociencias, Facultad de Química, UdelaR, Montevideo, Uruguay; McGill University, CANADA

## Abstract

Helminths express various carbohydrate-containing glycoconjugates on their surface, and they release glycan-rich excretion/secretion products that can be very important in their life cycles, infection and pathology. Recent evidence suggests that parasite glycoconjugates could play a role in the evasion of the immune response, leading to a modified Th2-polarized immune response that favors parasite survival in the host. Nevertheless, there is limited information about the nature or function of glycans produced by the trematode *Fasciola hepatica*, the causative agent of fasciolosis. In this paper, we investigate whether glycosylated molecules from *F*. *hepatica* participate in the modulation of host immunity. We also focus on dendritic cells, since they are an important target of immune-modulation by helminths, affecting their activity or function. Our results indicate that glycans from *F*. *hepatica* promote the production of IL-4 and IL-10, suppressing IFNγ production. During infection, this parasite is able to induce a semi-mature phenotype of DCs expressing low levels of MHCII and secrete IL-10. Furthermore, we show that parasite glycoconjugates mediate the modulation of LPS-induced maturation of DCs since their oxidation restores the capacity of LPS-treated DCs to secrete high levels of the pro-inflammatory cytokines IL-6 and IL-12/23p40 and low levels of the anti-inflammatory cytokine IL-10. Inhibition assays using carbohydrates suggest that the immune-modulation is mediated, at least in part, by the recognition of a mannose specific-CLR that signals by recruiting the phosphatase Php2. The results presented here contribute to the understanding of the role of parasite glycosylated molecules in the modulation of the host immunity and might be useful in the design of vaccines against fasciolosis.

## Author Summary

*Fasciola hepatica* is a helminth that infects mainly ruminants, causing great economic losses worldwide. Importantly, fasciolosis is also considered an emerging zoonosis with an increasing number of human infections globally. As other helminths, *F*. *hepatica* is able to regulate the host immune response favoring parasite survival in the host. In this work we investigated whether glycoconjugates produced by this parasite play a role in the host immune-regulation. Glycans, composed by carbohydrate chains, participate in important biological processes, but their role during *Fasciola* infection has not been previously addressed. We found that glycoconjugates are involved in the production of the regulatory cytokine IL-10 and in the production of the Th2-like cytokines IL-4. Furthermore, we found that they are also involved in the modulation of dendritic cell maturation, the most efficient antigen presenting cells. Indeed, the parasite is able to inhibit the maturation of dendritic cells in a process that is glycan-mediated and dependent on a mannose-specific receptor. In conclusion, our results highlight the importance of parasite glycoconjugates in the modulation of host immunity and might be applied in the design of vaccine strategies to prevent infection.

## Introduction

Fasciolosis is a major parasitic disease of livestock that causes significant economic losses worldwide [1–2]. Currently, fasciolosis is also considered an emerging zoonosis with an increasing number of human infections globally [1]. In temperate regions this disease is caused by the liver fluke *Fasciola hepatica*. During infection, this pathogen can modulate the host immune response by different cellular and molecular mechanisms that include the production of immune-suppressive cytokines by the host [3], the increase of regulatory T cells [4], the alternative activation of macrophages [3] or the modulation of maturation and function of dendritic cells (DCs) [5–7].

Helminths express various carbohydrate-containing glycoconjugates on their surface and they release glycan-rich excretion/secretion products that can be very important in their life cycles and pathology, since they can participate in immune escape [8]. Carbohydrate-signatures from parasites are decoded by the immune system through the interaction of several immune receptors. In particular, receptors of innate immunity that recognize glycan motifs consist of soluble or membrane-associated lectins, siglecs and scavenger receptors, among others. Notably, C-type lectin receptors (CLRs) have been described to mediate internalization of parasite glycosylated molecules as well as cell-surface signaling, modulating the host immune response [9]. For instance, *Schistosoma mansoni*, through a glycosylated RNAse, impairs protein synthesis of IL-12. The glycans on this enzyme are essential to allow its uptake by DCs where it degrades both ribosomal and messenger RNA, leading to a Th2-polorized T-cell response [10]. On the other hand, glycans from the nematode *Brugia malayi* were reported to participate in the induction of the specific Th2 immune response, since sodium periodate-treated soluble extracts from this parasite induced lower levels of IL-4 by specific lymph node cells [11].

Evidence demonstrating that helminths can mediate the modulation of the activity or function of DCs has also been reported [5–7]. DCs are potent antigen presenting cells that possess the ability to stimulate naive T cells. In response to infectious agents DCs undergo a maturation process during which they migrate to secondary lymphoid organs where they present captured antigens to naive T cells, for the triggering of specific immunity. This process is associated to an up-regulation of the expression of MHC molecules, adhesion molecules and co-stimulatory molecules (CD40, CD80 or CD86) as well as a down-regulation of their endocytic capacity [12]. However in the presence of helminth antigens mature DCs express reduced levels of co-stimulatory markers and MHC class II molecules, as compared to DCs matured with Toll-like receptor (TLR) ligands such as lipopolysaccharide (LPS) [13]. Also, these DCs are not capable of producing high levels of pro-inflammatory cytokines (IL-12, IL-6 or TNFα) [13]. In this sense, independent *in vitro* studies have reported that different *F*. *hepatica* components modulate TLR-initiated DC maturation and their stimulatory function [5–7].

Although under investigation, the identity of the molecular components from helminths that mediate DC immune-modulation is limited. Nevertheless, growing evidence suggests that parasite glycoconjugates could play a role in the modulation of DC-maturation [14]. Indeed, a recent report described that glycosylated components from the whipworm *Trichuris suis* mediate the suppression of TNFα production by DCs stimulated with LPS, through the recognition of mannose (Man) residues or terminal N-acetyl-Galactosamine (GalNAc) by specific CLRs [15]. Interestingly, it has been recently reported that fucose-carrying helminth components can trigger a DC-SIGN specific signaling pathway on DCs that directs differentiation of T cells into follicular helper T cells [16].

Little is known about the glycans produced by *F*. *hepatica*, with only two recent reports describing lectin reactivity in the miracidial surface [17] or in the gut of adult flukes [18–19], that suggest the presence of Man and glucose (Glc) residues. Another independent work from Wuhrer and collaborators described Galβ1-6Gal-terminating glycolipids by using mass spectrometry [20]. Finally, our group has previously described the expression of the GalNAc-*O*-Ser/Thr structure (known as Tn antigen) [21]. As for their structure, the immune-modulatory roles of *F*. *hepatica* glycans have also barely been investigated. Their role in alternative activation of macrophages has been reported by treating glycans with periodate [3] or by inhibiting macrophage binding and function using antibodies specific for CLRs [22–23]. Nevertheless, the evidence available about the function of *F*. *hepatica* carbohydrate in the regulation of parasite immunity or DC function is still poor.

In this work, we show that glycoconjugates from *F*. *hepatica* are involved in the modulation of host immunity, promoting the production of IL-4 and IL-10, and suppressing IFNγ production. During infection this parasite is able to induce a semi-mature phenotype of DCs which express low levels of MHCII and secrete IL-10. Furthermore, we show that parasite glycosylated molecules mediate the modulation of LPS-induced maturation of DCs since their oxidation restores the capacity of LPS-treated DCs to secrete high levels of the pro-inflammatory cytokines IL-6 and IL-12/23p40 and low levels of the anti-inflammatory cytokine IL-10. Inhibition assays using carbohydrates suggest that the immune-modulation is mediated by the recognition of a Man specific-CLR that signals by recruiting the phosphatase Php2. The results presented here contribute to the understanding of the role of parasite glycoconjugates in the modulation of the host immunity and might be useful in the design of vaccines against fasciolosis.

## Methods

### Ethics statement

Mouse experiments were carried out in accordance with strict guidelines from the National Committee on Animal Research (Comisión Nacional de Experimentación Animal, CNEA, National Law 18.611, Uruguay). Adult worms were collected during the routine work of a local abattoir (Frigorífico Carrasco) in Montevideo (Uruguay). All procedures involving animals were approved by the Universidad de la República's Committee on Animal Research (Comisión Honoraria de Experimentación Animal, CHEA Protocol Numbers: 071140-001822-11 and 071140-000143-12).

### Mice

Six- to 8-week-old female BALB/c mice were obtained from DILAVE Laboratories (Uruguay). Animals were kept in the animal house (URBE, Facultad de Medicina, UdelaR, Uruguay) with water and food supplied *ad libitum*, and handled in accordance with institutional guidelines for animal welfare by the Committee on Animal Research (CHEA, Uruguay).

### Preparation of protein lysates from *F*. *hepatica*

Live adult worms of *F*. *hepatica* were obtained from the bile ducts of bovine livers, washed in phosphate buffered saline (PBS) pH 7.4, then mechanically disrupted and sonicated. After centrifugation at 40,000 × *g* for 60 min supernatants were collected and dialyzed against PBS. The obtained lysate (FhTE) was resuspended on PBS containing a cocktail of protein inhibitors (Sigma-Aldrich, St. Louis, MO) and dialyzed against PBS for 24 h. Carbohydrate glycol groups present in FhTE were oxidized with sodium periodate (10 mM). The oxidation was performed at room temperature for 45 min in the dark, followed by the reduction with sodium borohydride (50 mM) of the reactive aldehyde groups. The resulting oxidized lysate is referred as FhmPox. In order to perform control experiments, the following control extracts were prepared: FhCB, consisted of FhTE subjected to the whole treatment excepting for the incubation with sodium periodate; and CmPox, consisting of PBS subjected to the entire treatment. Lysates were dialyzed against PBS and their protein concentration was measured using the bicinchoninic acid assay (Sigma-Aldrich, St. Louis, MO). To remove endotoxin contamination, the lysates were applied to a column containing endotoxin-removing gel (detoxi-gel, Pierce Biotechnology). The endotoxin levels were determined by using the Limulus Amebocyte Lysate kit Pyrochrome (Associates of Cape Cod). Protein preparations showed very low levels of endotoxins and were not able to induce DC maturation (as IL-12 read out) on their own. The concentration of all *F*. *hepatica* extracts described here and used in culture experiments did not modify cell viability evaluated by MTT (2-[4,5-dimethyl-2-thiazolyl]-3,5-diphenyl-2H-tetrazolium bromide) assay.

### Electrophoresis and western blot of parasite lysates

The lysates were analyzed by electrophoresis and western blotting using the anti-Tn mAb 83D4 (kindly provided by E. Osinaga, Uruguay) and a polyclonal antibody specific for the Cathepsin-L1 from *F*. *hepatica* (FhCL1, kindly provided by P. Berasain, Uruguay). Proteins were separated in a 15% SDS-PAGE and transferred to nitrocellulose sheets (Amersham, Saclay, France) at 45 V overnight in 20 mM Tris–HCl, pH 8.3, 192 mM glycine and 10% ethanol. Residual protein-binding sites were blocked by incubation with 1% bovine serum albumin (BSA) in PBS at 37°C for 1 h. The nitrocellulose was then incubated for 2 h at room temperature with either the anti-Tn mAb 83D4 or the anti-FhCL. After three washes with PBS containing 0.1% Tween-20, the membrane was incubated for 1 h at room temperature with an anti-mouse or anti rabbit immunoglobulins, conjugated to peroxidase (Dako, CA, USA) diluted in PBS containing 0.1% Tween-20 and 0,5% BSA and reactions were developed with enhanced chemiluminiscence (ECL) (Amersham, Saclay, France). The same procedure was performed omitting the primary antibodies as a negative control.

### Infections and cell cultures

BALB/c mice of 8 weeks old (5 per group) were orally infected with 10 uncapped *F*. *hepatica* metacercariae (Baldwin Aquatics, USA) per animal. After 1, 2 or 3 weeks of infection spleens, hepatic draining lymph nodes (HLN) and peritoneal exudates cells (PECs) were removed. PECs were harvested by washing the peritoneal cavity with 10 mL of cold PBS. Splenocytes, HLN, PECs (0.5–1 × 10^6^ cells/mL) or purified CD4^+^ T cells (0.2 × 10^6^ cells/mL) were cultured in complete medium consisting of RPMI-1640 with glutamine (PAA Laboratories, Austria) supplemented with 10% heat-inactivated fetal bovine serum (FBS), 50 μM 2-mercaptoethanol, 100 U/ml penicillin and 100 mg/ml streptomycin (Sigma-Aldrich, St. Louis, MO), in the presence or absence of FhTE (75 μg/ml), Concavalin-A (ConA) (5 μg/ml), FhmPox (75 μg/ml) or the controls FhCB and CmPox, for 72 h at 37°C and 5% CO_2_. IFNγ, IL-4, IL-5 and IL-10 levels were evaluated by specific ELISAs or quantitative RT-PCR (qRT-PCR). Uninfected naive animals were used as a control group. Proliferation with the control CmPox always provided background levels, such as with medium alone. Infections were also carried with 5, 10 or 15 metacercariae/mouse and mice were sacrificed at 3 weeks after the infection. Alanine transaminase activity was measured in sera from infected and non-infected animals using a commercial kit (Spinreact, Spain), according to the manufacturer’s instructions. The presence of flukes in livers from infected animals was analyzed so as to calculate fluke burden. Livers from infected mice presented macroscopic damage and/or necrosis. They were also histologically analyzed and found to present inflammatory infiltration and flukes, in some cases.

### Determination of cytokines and chemokines

IFNγ, IL-4, IL-5, IL-6, IL-10 and IL-12p40/23 levels on culture supernatants were quantified by interleukin-specific sandwich ELISA assays (BD Bioscience, NJ, USA). MIP-1α and MIP-2 were also detected in the culture media using sandwich ELISA assays, according to the instructions of the manufacturer (RayBiotech, Inc., GA, USA). In some cases, cytokines were detected by qRT-PCR using a Corbett Rotor Gene 6000 Real-Time PCR Machine and the SYBR Green 1 dye (Applied Biosystem). Standard amplification conditions were 3 min at 95°C and 40 cycles of 10 s at 95°C, 30 s at 60°C, and 30 s at 72°C. For detection of cytokines the following primers were used: *IFNγ*: F: 5′-GGAGGAACTGGCAAAAGGATGGTGA-3′ and R: 5′-GCGCTGGACCT-GTGGGTTGT-3′; *IL-4*: F: 5′-AGGTCACAGGAGAAGGGACGCC-3′ and R: 5′-TGC-GAAGCACCTTGGAAGCCC-3′; *IL-10*: F: 5′-TTCCCAGTCGGCCAGAGCCA and R: 5′-GGGGAGAAATCGATGACAGCGCC-3′. Results were expressed as the ratio between each evaluated cytokine and *GAPDH* expression. For *GAPDH* detection, sense and antisense primers were 5′-TCGGAGTCAACGGATTG-3′ and 5′-CCTGGAAGAT-GGTGATGG-3′, respectively.

### Evaluation of antibody reactivity

The reactivity of polyclonal antibodies from infected or non-infected animals was evaluated by ELISA. Briefly, ninety-six-well microtiter plates (Nunc, Roskilde, Denmark) were coated overnight at 4°C with 1 μg/well of FhTE in 50 mM carbonate buffer (pH 9.6). After blocking with 1% BSA in PBS, three washes with PBS containing 0.1% Tween-20 were performed. For the oxidation of the glycan moieties of FhTE, wells were treated with 10 mM of sodium meta-periodate in 50 mM sodium acetate buffer pH 4.5 for 30 min at room temperature in the dark, washed with 50 mM sodium acetate buffer and subsequently incubated for 1 h with glycine 1% at room temperature. As controls (Fhmock), wells were subjected to the same treatment except for the incubation with sodium meta-periodate. Serially diluted sera in buffer (PBS containing 0.1% Tween-20 and 0.5% BSA) were added to the wells for 1 h at 37°C. Following three washes, wells were treated 1 h at 37°C using goat anti-mouse polyvalent peroxidase-conjugate (Sigma-Aldrich, St. Louis, MO) and *o*-phenylenediamine-H_2_O_2_ was then added as substrate. Plates were read photometrically at 492 nm in an ELISA auto-reader (Labsystems Multiskan MS, Finland).

### Lectin recognition of FhTE

The lectin-reactivity on FhTE, FhmPox or FhCB lysates was evaluated by an ELISA-type assay. Briefly, Nunc microtiter plates were coated with 2.5 μg/well of parasite lysates and blocked with 1% BSA in PBS for 1 h at 37°C. Then, different concentrations of biotin coupled lectins were added and incubated for 1 h at 37°C. For inhibition assays, the lectins were pre-incubated for 30 min at 37°C with 50 mM of the indicated monosaccharide. After three washes, streptavidin conjugated to DyLight 800 was added to each well for 30 min at 37°C. Plates were then washed and analyzed with an Odyssey Infrared Imaging System (LI-COR Biosciences, NE, USA). Lectins from *Vicia villosa* (VV: GalNAc, Tn antigen), *Triticum vulgaris* (WGA: (GlcNAc)_2_), *Canavalia ensiformis* (ConA: αMan>αGlc), *Arachis hpogaea* (PNA: βGal(1,3)GalNAc), *Ulex europaeus* (UEA: Fucα(1,2)Gal), *Erythrina cristagalli* (ECA: βGal(1–4)GlcNAc), *Sambucus nigra* (SNA: αNeuAc(2,6)Gal) and *Helix pomatia* (HPM: GalNAc) were used in this study.

### Cell analyses by flow cytometry

Splenocytes or PECs from infected and non-infected mice were washed twice with PBS containing 2% FBS and 0.1% sodium azide. Cells were then stained with different antibody mixes to identify DCs or macrophages. First, CD3^+^ cells were excluded from the gatings. DCs were defined as CD11c^hi^ F4/80^-^ CD3^-^ cells. Macrophages were identified as F4/80^+^ CD11c^-^ CD3^-^ cells. The following antibodies were used in these experiments: anti-CD3 (17A2), CD11c (N418), CD40 (HM40-3), I-A/I-E (2G9), F4/80 (BM8), CD80 (16-10A1), CD86 (GL1). Cells were then washed twice with PBS containing 2% FBS and 0.1% sodium azide and fixed with 1% formaldehyde. Cell populations were analyzed using a CyAn ADP Analyzer (Beckman Coulter). Antibodies were obtained from Affymetrix (CA, USA) or from BD-Biosciences (CA, USA). IL-10 and IL-12/IL23p40 *in vivo* production by DCs or macrophages was analyzed by intracellular staining. Splenocytes and PECs from infected and non-infected mice were cultured for 6 h with GolgiPlug (BD Biosciences), washed, stained with CD11c, F4/80 and CD3, and then fixed and permeabilized using the Cytofix/Cytoperm kit (BD Biosciences) and subsequently stained with Abs specific for IL-12/23p40 or IL-10 (Biolegend, CA, USA).

### Dendritic cell generation and maturation

Bone Marrow-derived Dendritic Cells (BMDCs) were generated from bone marrow precursors from BALB/c mice. Briefly, bone marrow precursor cells were harvested and plated at a density of 2–5 × 10^5^ cells/ml in complete culture medium supplemented with GM-CSF-containing supernatant. After 3 days of culture at 37°C, the medium was replaced. Cells were recovered on day 8 and analyzed for the expression of CD11c by flow cytometry. To analyze DC-maturation, BMDCs (2.5 × 10^5^/well) were incubated at 37°C and 5% CO_2_ in 96-well plates with FhTE, FhPox, FhCB (75 μg/ml) or medium alone in the presence or absence of LPS (*Escherichia coli* 0111:B4, 0.5–1 μg/ml) overnight at 37°C. Alternatively, cells were pre incubated for 45 min. at 37°C with 10 mM of monosaccharides (Man, GalNAc or arabinose) or 10 μM of specific signaling inhibitors (PHPS1; GW5074; and ER27319). Cells were then centrifuged at 1,500 rpm for 5 min at 4°C and supernatants were then collected. Cytokine (IL-12/23p40, IL-10 and IL-6) levels were tested on culture supernatants by interleukin specific sandwich ELISA assays (BD Bioscience, NJ, USA).

### Evaluation of the binding and uptake of parasite molecules by BMDCs

The *in vitro* internalization and binding of the lysates were analyzed by flow cytometry. BMDCs were incubated (2.5 x 10^5^/well) with Alexa 647-labeled Ag for 1 h at 37°C in complete medium (to assess uptake), or at 4°C in complete medium (to assess binding). Cells were then washed twice and analyzed by FACS. For inhibition assays, cells were preincubated with 5 mM EDTA or 50 mM of different carbohydrates for 30 min at 37°C.

### Statistical analysis

The Student t test was used for statistical comparisons; p values <0.01 or <0.05 were considered to be statistically significant, depending on the experiment.

## Results

### *Fasciola hepatica* induces strong levels of IL-4, IL-5 and IL-10 and suppresses IFNγ production

**Fig 1:**
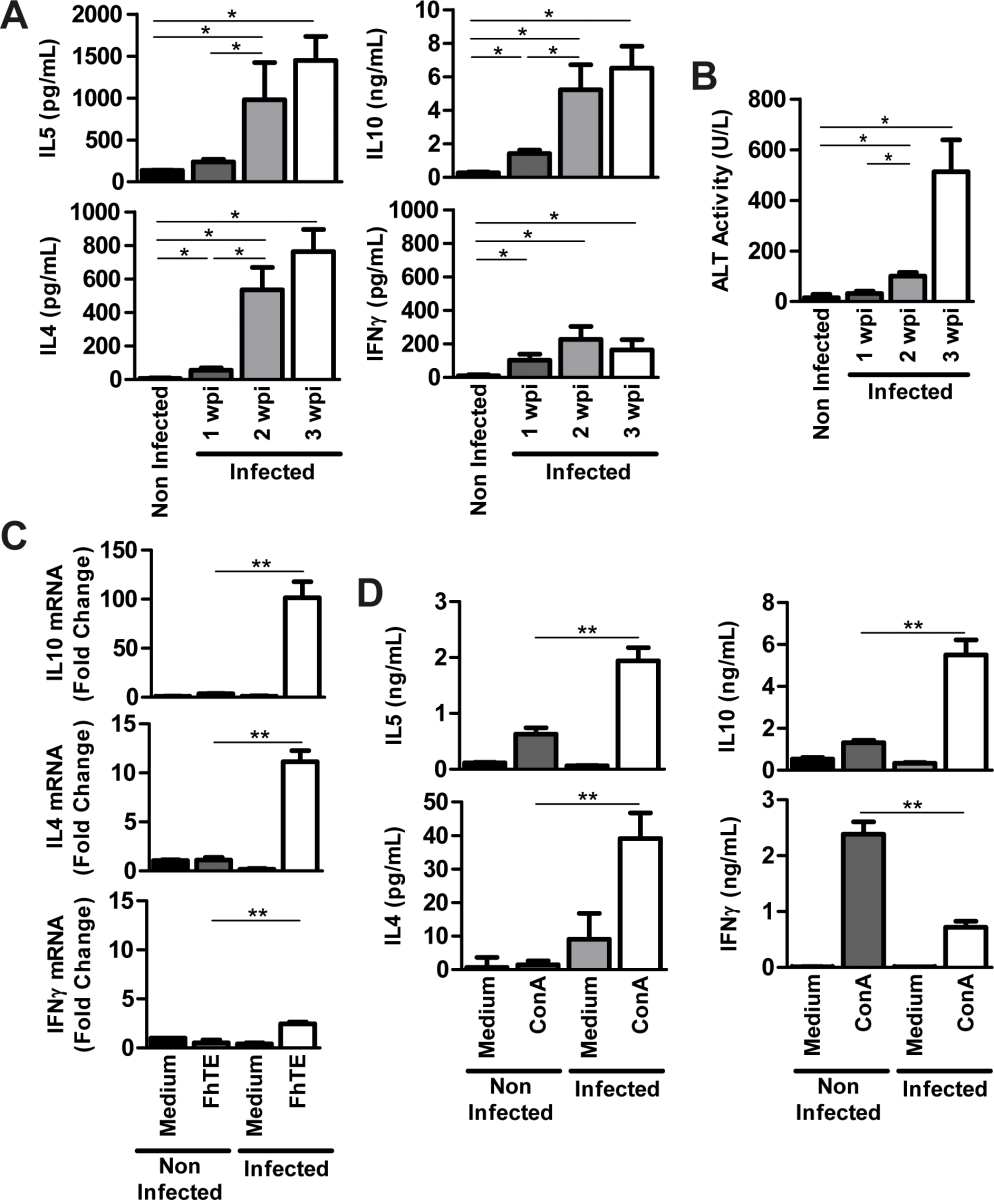
*F*. *hepatica* induces a strong modified-Th2 specific cellular immune response characterized by high levels of IL-4, IL-5 and IL-10. BALB/c mice (n = 5 per group) were orally infected with 10 metacercariae in PBS (infected mice). PBS alone served as a control (non-infected mice). Mice were bled and sacrificed one, two and three weeks after the infection and spleens were removed. (**A**) Splenocytes were cultured in the presence of FhTE (75 μg/mL) for 3 days at 37°C. Culture supernatants were collected and analyzed by ELISA for IL-4, IL-5, IL-10 or IFNγ. (**B**) Alanine transaminase activity was measured in sera from infected and non-infected. (**C**) Cytokines were also detected by quantitative RT-PCR. Results are shown as the ratio of mRNA amplification for a specific cytokine and the GAPDH. (**D**) Splenocytes from infected animals sacrificed at 3 wpi and non-infected mice were also stimulated with ConA (5 μg/mL) and cytokines on the supernatants were determined by ELISA. Results are expressed as the mean of three independent experiments (±SD, indicated by error bars). Asterisks indicate statistically significant differences (**p < 0.01; *p < 0.05) with respect to FhTE- or ConA-stimulated splenocytes from non-infected animals.

One of the objectives of this work was to evaluate the role of *F*. *hepatica* glycoconjugates structures in the induction of the host immune response. Indeed, the parasite induces a Th2 immune response with a regulatory component [3]. However, the parasite molecules involved in this immune-regulation are still unknown. Thus, we first evaluated different parameters of the immunity against *F*. *hepatica* in our experimental model and correlated them with the course of the infection and the level of liver damage. To this end, BALB/c mice were infected with 10 metacercariae and the production of IL-4, IL-5 IL-10 and IFNγ was evaluated on splenocytes from infected animals at 1, 2 and 3 weeks post-infection (wpi). Splenocytes removed from infected animals and stimulated *in vitro* with a total parasite extract (FhTE) produced high levels of IL-4, IL-5 and IL-10, with significant higher levels of IL-4 and IL-10 as soon as the first wpi, reaching a plateau at week 2 after infection. On the other hand, IFNγ was slightly increased, although at very low levels (≤200 pg/ml), compared to the 30-fold increase of IL-10 (around 6 ng/ml) (Fig 1A). The increase of IL-4 and IL-5 as well as the regulatory cytokine IL-10 coincided with the detection of liver damage evaluated by the alanine aminotransferase (ALT) activity in serum, a common marker to detect hepatic dysfunction [24]. Indeed, the ALT activity augmented 5-fold at 2 wpi, while it increased more than 20 fold at 3 wpi compared to levels detected in naïve/control animals (Fig 1B). ALT activity also augmented with parasite dose of infection, confirming its usefulness for detecting liver damage and monitoring *F*. *hepatica* infection (S1A Fig). The strong production of IL-4 and IL-10 at 3 wpi was confirmed by qRT-PCR, revealing around a 100-fold increase of IL-10 and only a 3-fold increase of IFNγ expression, with respect to uninfected animals (Fig 1C). The strong modified Th2 polarization observed was also evidenced with a polyclonal stimulus, such as ConA, on splenocytes from infected animals. They produced higher levels of IL-4, IL-5 and IL-10, while their capacity to produce IFNγ was significantly diminished (a 3-fold decrease), comparing to splenocytes from naïve animals stimulated in the same conditions (Fig 1D). These results are in agreement with previous work describing the Th2 polarization induced by *F*. *hepatica* during infection [3].

### *F*. *hepatica* produces glycosylated components carrying diverse carbohydrate moieties

**Fig 2:**
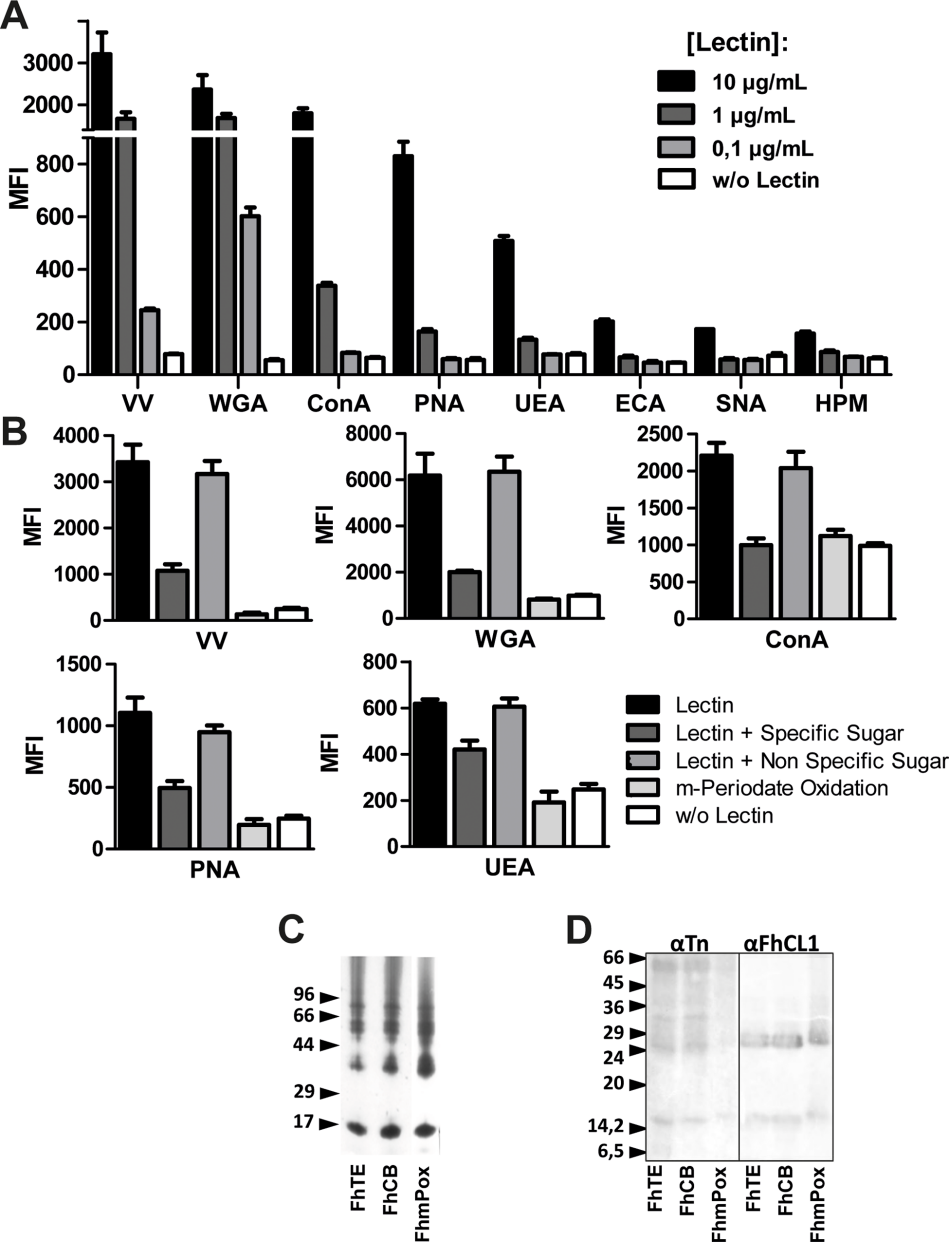
*F*. *hepatica* produces a diverse variety of glycan structures. **(A)** Lectin reactivity was evaluated on microplates coated with FhTE (2.5 μg/well) using different concentrations of biotin-conjugated lectins and streptavidin-Dylight-800. Mean fluorescence intensity (MFI) was determined on plates with an infrared imaging system. Lectins used in this study were: VV (GalNAc, Tn antigen), WGA ((GlcNAc)_2_), ConA (αMan>αGlc), PNA (βGal(1–3)GalNAc) and UEA (Fucα(1–2)Gal), ECA (βGal(1–4)GlcNAc), SNA (αNeuAc(2–6)Gal) and HPM (GalNAc). **(B)** Carbohydrate specificity was demonstrated by performing inhibition assays with specific carbohydrates (50 mM) by pre-incubating with GalNAc (VV), GlcNAc (WGA), Man (ConA), Gal (PNA) or Fuc (UEA). Alternatively, lectin reactivity was evaluated on periodate-oxidized glycans (FhmPox) or control consisting of FhTE only treated with borydrure (FhCB). **(C)** Parasite lysates were subjected to SDS-PAGE (15%) and stained with silver nitrate. **(D)** Alternatively, they were transferred to PVDF membranes and incubated for 2 h at RT with the anti-Tn monoclonal antibody 83D4 or a polyclonal anti-cathepsin L1 serum.

Carbohydrate structures produced by parasites participate in critical processes such as infection or invasion [25–26]. Although much advance in the area of glycomics has been gained in recent years, the knowledge about the structure and function of *F*. *hepatica* glycans is still poor. In order to identify the carbohydrates present in the FhTE used in this study we carried out lectin-reactivity assays with a panel of different vegetal lectins. Glycoconjugates from FhTE strongly reacted with lectins from *Vicia Villosa* (VV), *Triticum vulgaris* (WGA), *Canavalia ensiformis* (ConA), *Arachis hpogaea* (PNA) and *Ulex europaeus* (UEA) (Fig 2A), revealing the presence of N-acetil-galactosamine-Ser/Thr (GalNAc-Ser/Thr), N-acetyl-glucosamine (GlcNAc)_2_, mannose (Man) or glucose (Glc), galactose (Gal) in (βGal(1–3)GalNAc and fucose (Fuc) in Fucα(1–2)Gal, respectively. We also performed inhibition assays with specific monocarbohydrates and with non specific carbohydrates as negative control. More than the 70% of the lectin reactivity was lost when incubating with GalNAc, GlcNAc or Man and VV, WGA or ConA, respectively, confirming the carbohydrate specificity by these lectins (Fig 2B).

Finally, we carried out the assays on oxidized FhTE (FhmPox). Mild periodate oxidation of glycans is usually used to evaluate the functional roles of glycoconjugates [11, 15]. During this process the glycol groups in carbohydrates are oxidized to reactive aldehyde groups, which are in turn reduced with sodium borohydride. Thus, the structure of carbohydrates is lost, as well as the possible biological activity that they can mediate. The recognition of the specific carbohydrates on FhTE by most reactive lectins was completely abrogated with meta-periodate oxidation (FhmPox) (Fig 2B). Importantly, the integrity of oxidized parasite glycoconjugates (FhmPox) remained unchanged since the electrophoretic mobility of FhTE molecular components was similar to that of FhmPox (Fig 2C). Furthermore, oxidation with meta-periodate totally abolished the recognition of a monoclonal antibody specific for the GalNAc-O-Ser/Thr carbohydrate structure that is reactive to FhTE [27], while it did not modify the recognition of cathepsin-L1 by a specific polyclonal antibody (Fig 2D). Thus, the chemical oxidation of terminal carbohydrates abrogates recognition of glycans by carbohydrate binding proteins, resulting in an adequate strategy to study their recognition by proteins or receptors and could be useful to study their biological functions.

### *F*. *hepatica* carbohydrates participate in the production of high levels of IL-4 and IL-10, and in the decrease of IFNγ

**Fig 3:**
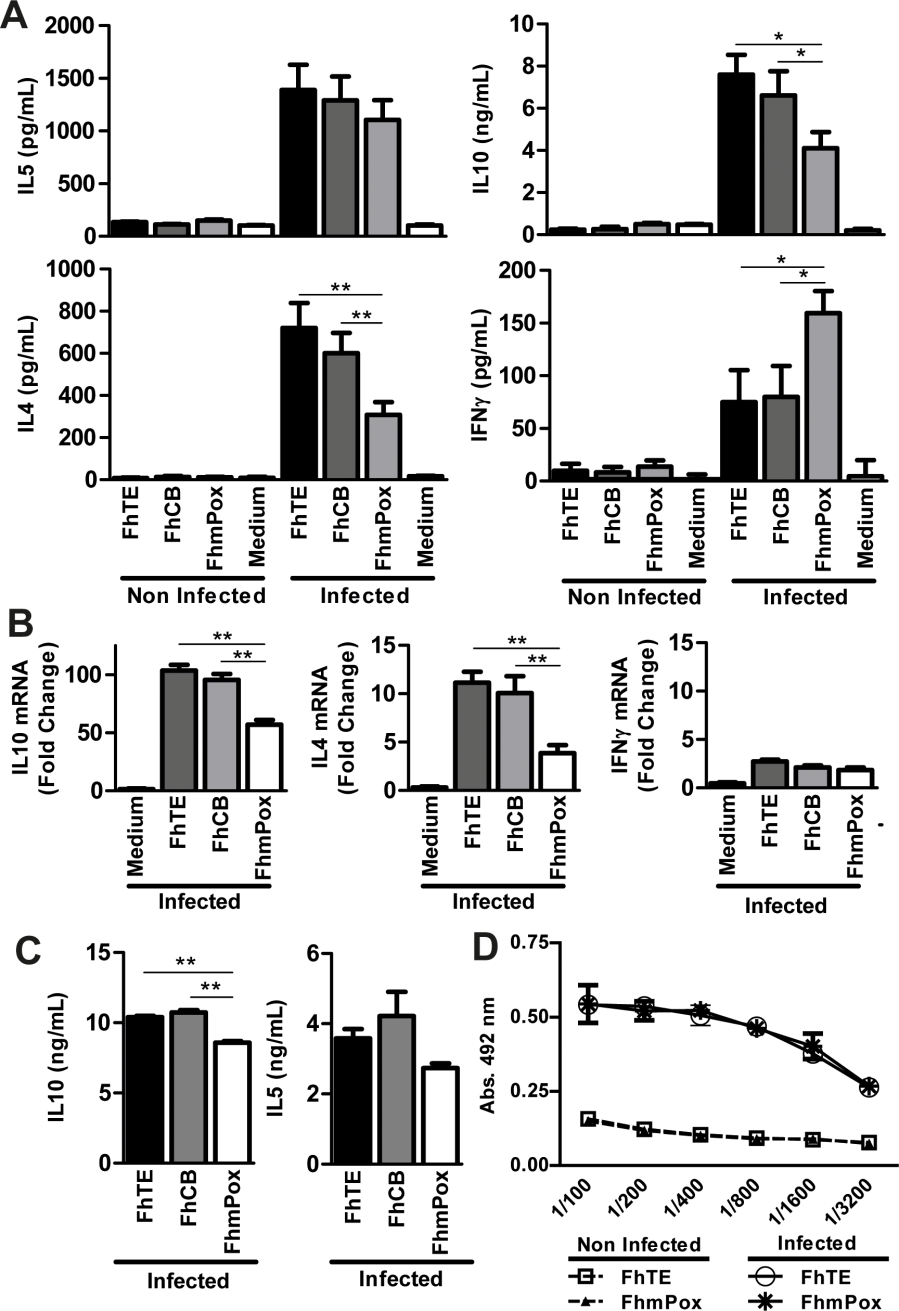
Oxidation of glycans results in a decreased production of IL-4 and IL-10 and an increase of IFNγ by parasite specific splenocytes. BALB/c mice (n = 5 per group) were infected with 15 metacercariae. After three weeks, animals were bled, sacrificed and spleens and hepatic draining lymph nodes were removed. Splenocytes were cultured in the presence of 75 μg/mL of FhTE, FhCB (oxidation negative control) or FhmPox (oxidized FhTE). Culture supernatants were collected and analyzed by ELISA for IL-4, IL-5, IL-10 or IFNγ (**A**). Alternatively, IL-4, IL-10 and IFNγ were detected by quantitative RT-PCR. Results are shown as the ratio of mRNA amplification for a specific cytokine and the GAPDH (**B**). Also, cells from hepatic draining lymph nodes were stimulated with FhTE, FhCB or FhmPox (75 μg/mL) and IL-5 and IL-10 on the supernatants were determined by ELISA (**C**). Total antibodies in sera from infected and non-infected animals specific for FhTE or FhmPox were evaluated on plates coated with parasite components (10 μg/ml) and with serial dilution of animal sera (**D**). Results are expressed as the mean of three independent experiments (±SD, indicated by error bars). Asterisks indicate statistically significant differences (**p < 0.01; *p < 0.05) with respect to FhTE-stimulated splenocytes from non-infected animals.

To determine whether *F*. *hepatica* glycosylated molecules participate in the induction of high levels of the Th2-type cytokines and the suppression of IFNγ we cultured splenocytes from infected animals with oxidized parasite components. Thus, splenocytes removed from infected animals at 3 wpi were *in vitro* stimulated with FhTE, and the production of cytokines in the culture supernatant was determined and compared to those incubated with oxidized parasite components (FhmPox). Splenocytes stimulated with FhmPox produced lower levels of IL-4 and IL-10 than cells incubated with FhTE. Surprisingly, in these conditions, spleen cells produced significant higher levels of IFNγ (Fig 3A). On the other hand, the levels of IL-5 remained unchanged. Splenocytes incubated with the control FhCB, consisting in FhTE subjected to the whole treatment excepting for the incubation with sodium periodate, behaved essentially as cells in presence of FhTE, as expected. The cytokine production was specific of parasite components since stimulated spleen cells from non-infected animals did not produce any of the evaluated cytokines. The oxidation-dependent decrease of IL-4 and IL-10 by splenocytes from infected animals was also confirmed by qRT-PCR, although no significant difference was found between the production levels of IFNγ by FhmPox-stimulated splenocytes (Fig 3B). Cells from the hepatic draining lymph nodes of infected animals also produced decreased levels of IL-10 when stimulated with oxidized parasite components, with no changes in the production of IL-5 (Fig 3C).

Finally, in order to establish a possible role of glyans in the recognition of parasite molecules by the humoral immune response, we evaluated the recognition of FhmPox by sera from infected animals. IgG antibodies from infected animals recognized in a similar manner both FhTE and FhmPox, suggesting that oxidation of terminal glycans does not modify the recognition of parasite components by antibodies induced during infection (Fig 3D).

### *F*. *hepatica* modulates dendritic cells in vivo by decreasing the levels of MHC class II on their surface and increasing their ability to produce IL-10

**Fig 4:**
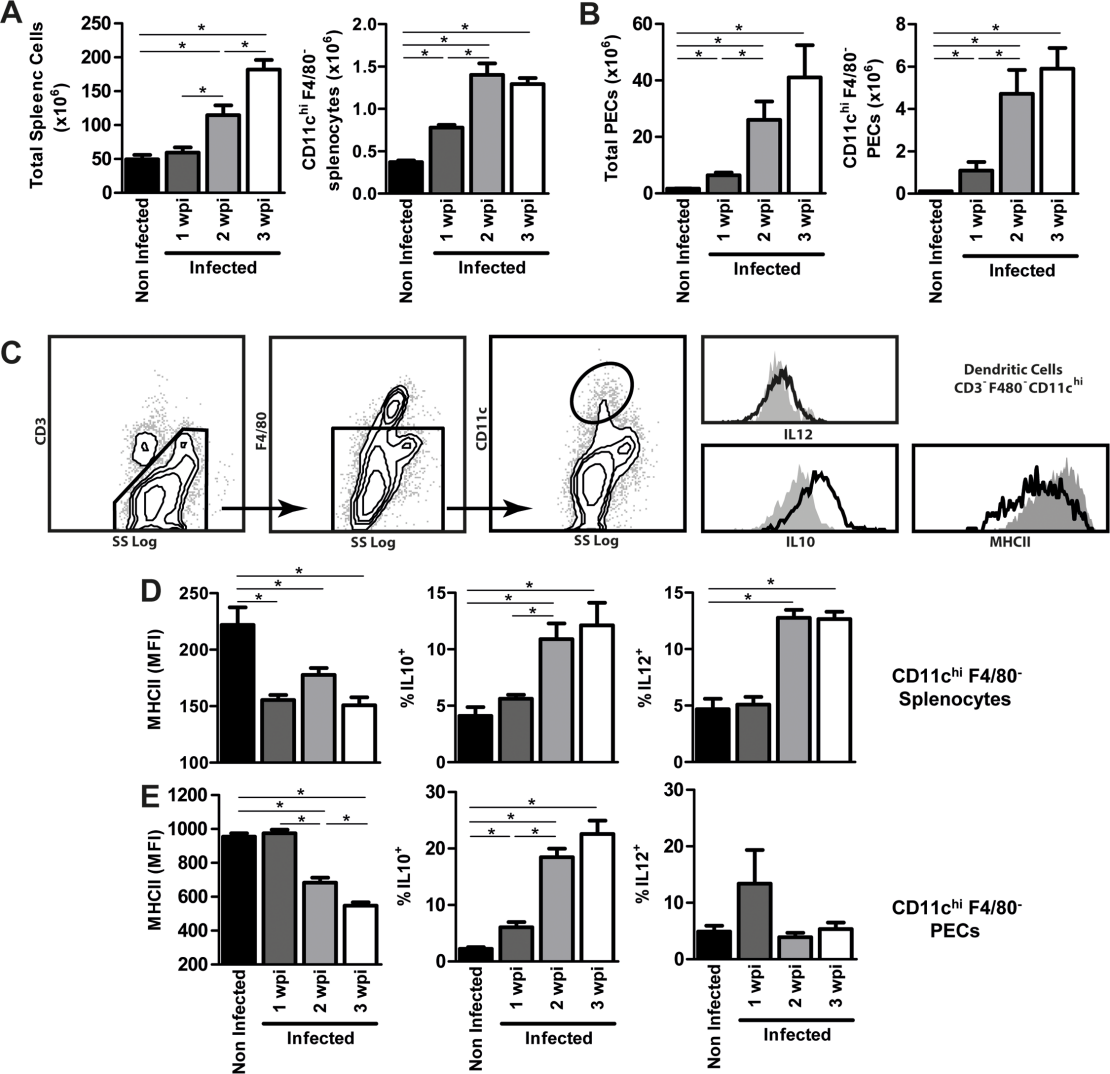
*F*. *hepatica* infection promotes the recruitment of MHC^low^ IL-10^+^ DCs both at the peritoneal cavity and spleen. Mice (n = 5 per group) were orally infected with 10 metacercariae in PBS (infected mice). PBS alone served as a control (non-infected mice). Mice were sacrificed one, two and three weeks after the infection and spleens and PECs were removed. Splenocyte (**A**) and PEC (**B**) suspensions were counted and the presence of CD11c^hi^ cells was analyzed by flow cytometry by staining cells with specific antibodies. CD11c^hi^ cells were selected after excluding CD3^+^ followed by exclusion of F4/80^+^ cells (C). Splenocytes (**D**) and PECs (**E**) were also incubated with anti-MCHII, permeabilized, and intracellularly stained with anti-IL-10 and IL-12/23p40 antibodies for 30 min at 4°C. Cells were analyzed on a flow cytometer. Results are expressed as the mean of three independent experiments (±SD, indicated by error bars). Asterisks indicate statistically significant differences (*p < 0.01) with respect to cells from non-infected animals.

Evidence demonstrating that *F*. *hepatica* components can modulate DC-maturation and function *in vitro* has been previously reported [5–6, 10, 28]. Also, the phenotype of DCs has been evaluated suggesting that the parasite immune-modulates DCs upon infection [13]. Nevertheless, an exhaustive study of DC immune-modulation by the parasite has not been carried out. Thus, in order to deeply evaluate their role in the host immune-modulation by *F*. *hepatica*, we sought to evaluate DCs *in vivo* both in the spleen and in the peritoneum of infected animals. Upon infection, we observed a marked recruitment of cells in the spleen and in the peritoneal cavity of infected animals, which increased with time of infection (Fig 4A and 4B, respectively). Among these cells, DCs (defined as CD11c^hi^ F4/80^-^ cells) were recruited both at the spleen and the peritoneum since the first wpi, and their number augmented with the course of infection (Fig 4A and 4B). In spite of the fact that all DCs were MHC class II positive, they presented remarkable decreased levels of MHCII expression (Fig 4C–4E). Indeed, splenic DCs presented around 50% reduction of MHCII expression since the first wpi together with lower levels of CD40 on their surface (S1B Fig). MHC class II- or CD40-decreased expression on DC surface was not modified when infecting animals with higher parasite dose (S1B Fig). Moreover, from the second wpi, an increase of IL-10 secreting splenic DCs was evidenced (Figs 4D and S2). Strikingly, IL-12^+^ DCs also augmented in the spleen (Fig 4D). DCs recruited into the peritoneum also reduced the expression of MHC class II (Fig 4E) while the expression of CD80 and CD86 was increased (S1C Fig). There was also an increase of IL-10 secreting DCs in the peritoneal cavity, while IL-12 secreting DCs remained very low in the peritoneum (Figs 4E and S2).

Macrophages, defined as F4/80^+^ CD11c^-^ cells were also recruited to the spleen and peritoneum upon infection (S3 Fig). In spleen, the recruitment of IL-10^+^ macrophages was favored while IL-12 secreting macrophages considerably decreased (S2A and S3C Figs). On the other hand, macrophages from the peritoneal cavity increased the expression of surface MHC class II from the first wpi. IL-10^+^ or IL-12^+^ macrophages were also increased in the peritoneum (S2 and S3D Figs).

### *F*. *hepatica* glycoconjugates modulate DC-specific stimulatory function

**Fig 5:**
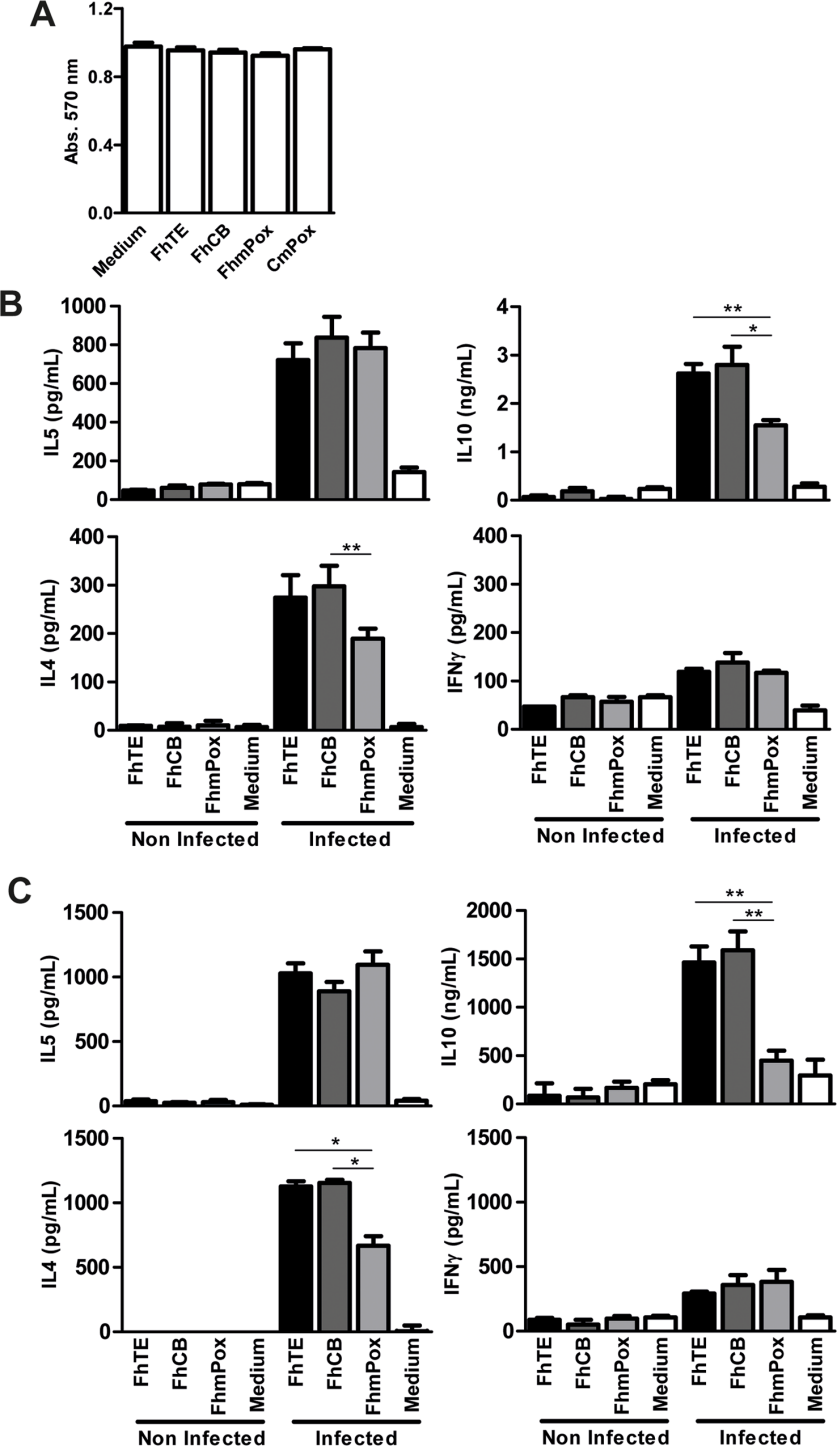
BMDCs pulsed with oxidized parasite lysate induce a decreased production of IL-4 and IL-10 by specific CD4^+^ T cells. BMDCs were cultured in the presence of 75 μg/mL of FhTE, FhCB (oxidation negative control) or FhmPox (oxidized FhTE) in the presence of LPS (1 μg/ml) overnight at 37°C. Then cell viability was evaluated by incubating with MTT for 4 h and reading absorbance at 570 nm (**A**). Alternatively, BMDCs were washed twice in complete medium and co-cultured for 3 days at 37°C with total splenocytes (**B**) or purified CD4^+^ T cells (**C**) from infected animals sacrificed after 3 wpi. Culture supernatants were collected and analyzed by ELISA for IL-4, IL-5, IL-10 or IFNγ Results are expressed as the mean of three independent experiments (±SD, indicated by error bars). Asterisks indicate statistically significant differences (**p < 0.01; *p < 0.05) with respect to FhTE/LPS-stimulated DCs.

In order to determine whether parasite glycan structures can also modulate the function of DCs, we evaluated the *in vitro* activation of splenocytes (Fig 5B) and purified CD4^+^ T cells (Fig 5C) from infected animals by DCs. To this end, bone marrow derived DCs (BMDCs) were loaded with parasite-derived components (FhTE) or with oxidized total lysate (FhmPox). A control consisting on FhTE subjected to the chemical process in absence of meta-periodate was also included (FhCB). Importantly, the viability of loaded-BMDC was not affected by the treatment with different parasite lysates (Fig 5A). Then, loaded BMDCs were washed and subsequently incubated with splenocytes or purified CD4^+^ T cells from infected animals. As seen in Fig 5B and 5C, when incubated with FhmPox-loaded BMDCs, splenocytes and purified CD4^+^ T cells produced lower levels of IL-4 and IL-10 than cells stimulated with FhTE-loaded BMDCs, while IL-5 and IFNγ production remained unchanged. No cytokine production was detected when loaded BMDCs were incubated with splenocytes or purified CD4^+^ T cells from uninfected animals (naïve) (Fig 5B and 5C). These results strongly suggest that glycoconjugates modulate DC-stimulatory capacity by increasing the production of IL-4 and IL-10 by CD4^+^ T cells during infection.

### *F*. *hepatica* components interact with and are uptaken by DCs via Man-specific CLRs

**Fig 6:**
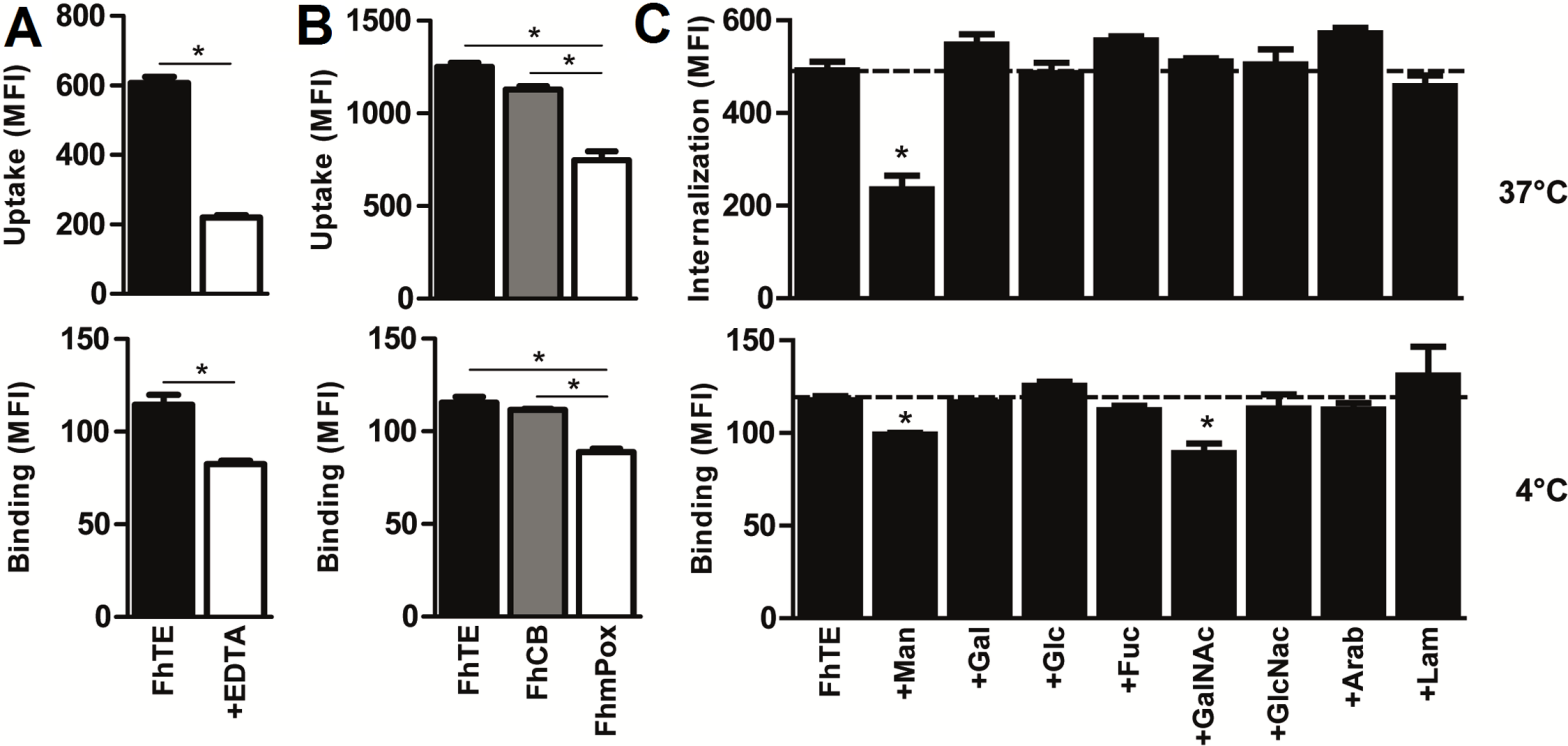
Glycoconjugates from *F*. *hepatica* interact with and are uptaken by BMDCs in a process mediated by Man-specific CLRs. BMDCs were incubated with Alexa 647-labeled FhTE for 1 h at 37°C in complete medium (to assess uptake), or at 4°C in complete medium (to assess binding) in presence or absence of EDTA (5 mM) and analyzed by flow cytometry on CD11c^+^ cells (**A**). Atto-labeled-FhTE, FhCB (control) or FhmPox (oxidized FhTE) were also incubated with BMDC and analyzed in the same conditions as in A (**B**). Inhibition assays with carbohydrates were carried out on BMDCs pre-incubated with 50 mM of different carbohydrates or laminarin for 30 min (**C**). Asterisks indicate statistically significant differences (*p* < 0.01) with respect to BMDC incubated with FhTE.

Glycans can modulate DC function through a variety of mechanisms. For instance, they can interact with lectin receptors expressed on the surface of DCs that can endocytose the glycan components and/or signal through kinase-dependent cascades [29]. Thus, we evaluated whether *F*. *hepatica* glycoconjugates could interact with the DC surface, or be internalized by DCs. To this end, Atto-647-labeled FhTE was incubated with DCs both at 4°C (to evaluate binding) or at 37°C (to test internalization) and the fluorescence intensity was determined by flow cytometry. As shown in Fig 6A and 6B, FhTE both interacted with and was internalized by DCs. To determine whether this binding or uptake was dependent on C-type lectin receptors, requiring Ca^2+^ for binding, we incubated DCs with FhTE in presence of EDTA, a chelating agent. We observed that around 70% of the FhTE internalization was abrogated with EDTA incubation, indicating that the internalization process was mediated by Ca^2+-^dependent lectin receptors (Fig 6A), On the contrary, only 25% of the binding was inhibited in presence of EDTA (Fig 6A), suggesting that CLR-dependent recognition of glycosylated molecules is less relevant in this process.

In order to confirm the participation of glycoconjugates in the internalization or binding of parasite components to DCs, FhCB and FhmPox were also stained with Atto-647 and further incubated with DCs in the same conditions as FhTE. Oxidation of parasite glycans resulted in a decrease of around 50% of FhTE internalization, while only 25% of reduction in the binding was observed (Fig 6B), indicating that they partially mediate binding and internalization of parasite components by DCs. As expected, no significant difference between FhTE and FhCB (control) binding or internalization was observed (Fig 6B).

Finally, we carried out inhibition assays with several carbohydrates, as well with laminarin, a ligand of the CLR Dectin-1 [30]. Laminarin was included in these assays since Dectin-1 seems to mediate the binding of *F*. *hepatica* glycoconjugates by macrophages [22]. As shown in Fig 6C, the binding of FhTE was inhibited by both Man and GalNAc, while only Man was capable of inhibiting FhTE uptake by DCs. Incubation with laminarin did not significantly modify FhTE binding or internalization by DCs, indicating that Dectin-1 is not involved in this process.

### *F*. *hepatica* modulates DC-maturation by increasing IL-10 and decreasing IL12/23p40 production through Man-specific CLRs

**Fig 7:**
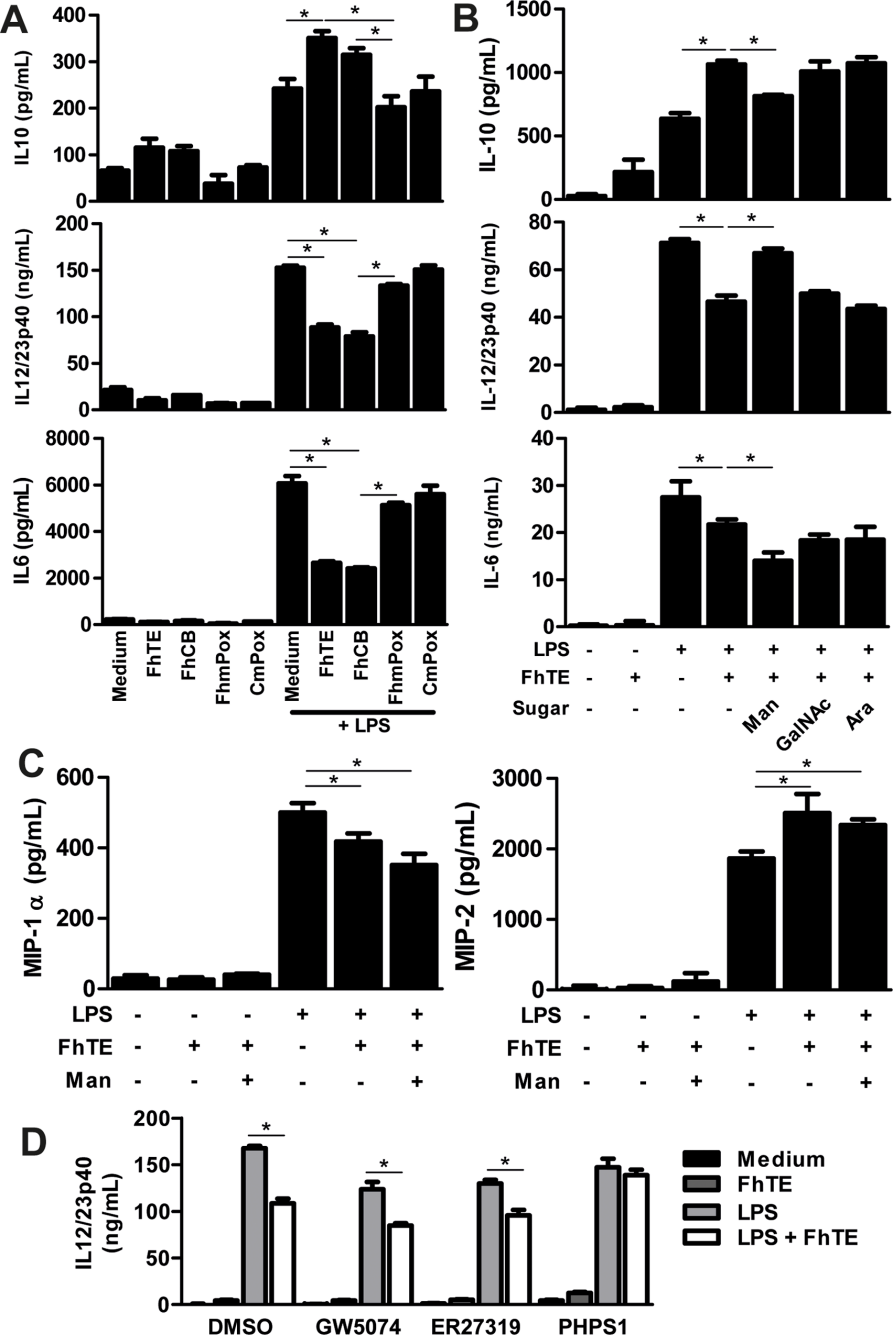
Oxidation of parasite components partially inhibits modulation of LPS-induced maturation of DCs by FhTE. BMDCs were cultured in the presence of 75 μg/mL of FhTE, FhCB (oxidation negative control) or FhmPox (oxidized FhTE) in presence or absence of LPS (1 μg/ml) overnight at 37°C. Then, culture supernatants were collected and analyzed by ELISA for IL-6, IL-10 or IL-12/23p40 (**A**). BMDCs were also pre-incubated for 45 min. with mannose (Man), N-Acetyl-Galactosamine (GalNAc) or arabinose (Ara) at 10 mM and then stimulated as in A. Culture supernatants were analyzed by ELISA for detection of IL-6, IL-10 or IL-12/23p40 (**B**) and MIP-1α and MIP-2 (**C**). Alternatively, BMDCs were pre-incubated for 45 min. with 10 μM of specific signaling inhibitors (PHPS1; GW5074; and ER27319) and then stimulated with FhTE (75 μg/mL) in presence of LPS (1 μg/ml) (**D**). Results are expressed as the mean of three independent experiments (±SD, indicated by error bars). Asterisks indicate statistically significant differences (*p < 0.01) with respect to LPS-stimulated BMDCs.

In order to evaluate whether parasite glycosylated molecules are able to influence DC-maturation we incubated BMDCs with parasite components or oxidized-FhTE in absence or presence of a maturation stimulus (LPS). Furthermore, the control FhCB (consisting in FhTE subjected to the whole treatment excepting for the incubation with sodium periodate) was also included. Then, we evaluated the production of several cytokines by DCs. BMDCs incubated with FhTE in presence of LPS produced higher levels of IL-10 than DCs incubated only with LPS. Interestingly, when oxidized-parasite glycans (FhmPox) where incubated with BMDCs, they produced similar levels of IL-10 than those produced by cells incubated with LPS alone, indicating that oxidation of glycans abrogates the immunomodulatory activity of FhTE on DCs (Fig 7A). On the other hand, the opposite situation was observed with the pro-inflammatory cytokines IL-6 and IL-12/23p40. Indeed, FhTE incubated with BMDCs in presence of LPS decreased the production of IL-6 and IL-12/23p40 induced by LPS, while FhTE oxidation restored the levels of both cytokines (Fig 7A). As expected, the control FhCB/LPS behaved essentially in the same way as FhTE/LPS, while the CmPox/LPS condition induced the same levels of cytokine production than cells incubated only with LPS. Altogether, these results indicate that glycoconjugates from *F*. *hepatica* modulate LPS-induced maturation of DCs by augmenting anti-inflammatory cytokines and decreasing pro-inflammatory cytokines.

Next, we carried out inhibition assays of the immunomodulatory capacity of FhTE with the carbohydrates Man and GalNAc since they were capable of inhibiting binding or internalization of FhTE. An irrelevant carbohydrate, arabinose (Ara) was used. As shown in Fig 7B, only Man could inhibit the immune-modulation of FhTE on DC-maturation. Indeed, incubation with Man restored the levels of IL-10 and IL-12/23p40 production induced by LPS alone on DCs. Nevertheless, the levels of IL-6 were not restored by inhibition assays with Man, suggesting that the production of this cytokine is triggered by a Man-independent signaling process. Incubation of DCs with FhTE in presence of GalNAc or Ara did not modify the levels of the evaluated cytokines (Fig 7B).

The production of the inflammatory chemokines MIP-1α and MIP-2 by DCs was also investigated. These chemokines regulate the influx of inflammatory cells, and can be produced by DCs under pathogenic conditions. MIP-1α is a ligand for CCR5, a chemokine receptor expressed on Th1 cells, while MIP-2 selectively chemoattracts Th2 cells [31]. DCs stimulated with LPS produced both chemokines. When incubated with FhTE the production of MIP-1α significantly decreased, while MIP-2 increased (Fig 7C). However, inhibition assays with Man did not modify the production of either chemokines induced by LPS/FhTE, suggesting that the production of these chemokines does not depend on Man-specific receptors on DCs.

Finally, in order to provide evidence about the possible CLR implicated in the recognition of Man-containing glycans from *F*. *hepatica*, we performed cell cultures in presence of chemical inhibitors of different signaling pathways: GW5074, ER27319 and PHPS1 that inhibit pathways mediated by Raf-1, Syk and Shp2, respectively. This inhibitors were chosen since a group of CLRs that recognize Man residues from pathogens, such as Dectin-1, Man Receptor, SIGNR or DCIR, are expressed on BMDCs and signal through these molecular mediators [29]. Thus, after incubation of these molecules, we evaluated the production of IL-12/23p40. Either GW5074 or ER27319 did not modify the decrease of IL-12/23p40 production induced by FhTE in presence of LPS. Nevertheless, incubation of DCs with PHPS1 in presence of FhTE/LPS completely abrogated the immunomodulatory effect of FhTE on DCs, leading to the production of similar levels of IL-12/23p40 as those obtained with LPS (Fig 7D).

## Discussion

Carbohydrate structures can exert different biological functions, ranging from cell growth or development to tumor growth or metastasis. Moreover, glycans participate in diverse processes such as coagulation, induction of immunity, cell-cell communication or microbial pathogenesis [32–33]. In this context, accumulating evidence demonstrates that glycoconjugates produced by helminths favor parasite survival by influencing the host immune response [34–35]. In this work we show that *F*. *hepatica* glycoconjugates are involved in the induction of high levels of IL-10 and IL-4 as well as in the reduction of IFNγ production by splenocytes during infection. In this sense, different previous reports used meta-periodate treatment of glycans to identify the role of glycoconjugates in the regulation of host immunity. Sodium meta-periodate treatment at low concentration does not remove glycans or compromise the integrity of glycoproteins, but instead opens up the “chair” structure altering molecular conformation of the glycans. Thus, when *F*. *hepatica* components were treated with meta-periodate and used to stimulate spleen cells from infected animals, they reduced their capacity to produce the Th2 cytokine IL-4 as well as the regulatory cytokine IL-10, suggesting a role of glycoconjugates in the induction of Th2/regulatory T cell immune response. Interestingly, stimulation of splenocytes from infected mice with oxidized parasite components also increased IFNγ production compared to the IFNγ levels obtained with *F*. *hepatica* total lysate containing non-modified glycans, indicating that they could suppress specific Th1 responses. Glycoconjugates from other helminths such as *Schistosoma mansoni* [36] and *Brugia malayi* [11] have also been reported to regulate the host immune shift toward a Th2 response, or inducing regulatory responses via induction of IL-10 [11, 37–38]. Indeed, *S*. *mansoni* produces the glycan structure Lewis^x^ (Galβ1-4(Fucα1–3)GlcNAc-terminal structure) that possesses immune-regulatory properties and accounts for induction of host IL-10 induced by the parasite [36]. *Fasciola hepatica*, however, does not express this glycan structure [39]. Thus, the immune-regulatory glycan structures from this parasite remain unknown.

Although carbohydrate moieties from parasites such as *Echinococcus* [40–42], *S*. *mansoni* [20, 43–45], among others [35, 46], have been well determined, glycoconjugates produced by *F*. *hepatica* still remained poorly characterized. Previous works have identified the presence of Gal(β1–6)Gal and GlcNAc(α1-HPO_3_-6)Gal terminating glycolipids by mass spectrometry [20, 47], as well as glycans carrying Man, Glc, GlcNAc or GalNAc by lectin reactivity [17–19, 48] both in tegument and tissues throughout the parasite. Here, we identified the presence of glycan structures containing Man/Glc, GalNAc or GlcNAc in the parasite lysate used in this study, confirming the strong binding of Man/Glc, GalNAc and GlcNAc-reactive lectins (ConA, WGA and VV, respectively). It is worth noting that, in our case, the lectin reactivity was abrogated when incubating with specific sugars, confirming the specificity for their respective carbohydrates. Our results also showed a slight recognition of parasite glycans by the *Ulex europeus* agluttinin (UEA-1) specific for terminal Fuc residues. However, it should be noted that the reactivity was lower than that observed with the other lectins, and that inhibition of lectin binding with Fucose did not completely abolish lectin recognition. Nevertheless, several helminths, including *F*. *hepatica*, are reactive to the *Lotus tetragonolobus* agglutinin, which binds Fucα1-3GlcNAc, confirming the expression of α1,3-fucosylated glycans [39]. In all cases, lectin reactivity was also abolished with meta-periodate oxidation of parasite glycans, demonstrating that this procedure suppressed carbohydrate specific recognition by lectins and is suitable for studying the biological role of glycans.

To evaluate possible mechanisms that could explain the modulation of host immunity by *F*. *hepatica* glycoconjugates we focused on DCs, the most effective antigen presenting cells that possess the ability to stimulate naive T cells, inducing a specific Th polarization [12]. In order to guarantee the identity of selected DCs, we excluded both macrophages and CD3^+^ cells and then selected CD11c^hi^ cells by flow cytometry analyses. Thus, DCs (CD3^-^ F4/80^-^ CD11c^hi^ cells) from infected animals presented a different phenotype than DCs from naïve animals, characterized by a profound decrease of MHC class II expression on DC-surface, which was not found in macrophages (CD3^-^ F4/80^+^ CD11c^-^ cells), probably due to a different interaction between these cells and parasite molecules. DCs recruited to the peritoneum also showed an up-regulation of the co-stimulatory molecules CD80 and CD86, suggesting that these DCs acquire a semi-mature phenotype upon parasite infection. The expression of MHC class II-peptide complexes on the surface of DCs is essential for their ability to activate CD4^+^ T cells efficiently. Apparently, *F*. *hepatica* would restrict the capacity of DCs to present antigens to CD4^+^ T cells as well as to prime specific CD4^+^ T cells, an effect that increases with persistence of infection. It has been previously shown that ubiquitination of MHCII-peptide complexes regulates their surface expression, retention and degradation in DCs [49–51], and that certain pathogens, such as *Salmonella typhimurium*, induce polyubiquitination of HLA-DR, resulting in a reduced surface expression of all MHC class II isotypes [52]. On the other hand, there are evidences reporting that *Mycobacterium tuberculosis* diminishes MHC-II synthesis by macrophages [53] in a process dependent on TLR2 ligation [54], limiting antigen presentation. It would be interesting to evaluate whether any of these molecular mechanisms underlie the reduced expression of MHC class II on the surface of DCs from *F*. *hepatica*-infected animals. There are also pieces of evidence highlighting the role of IL-10 in inducing immune anergy by reducing expression of MHC class II on the surface of antigen presenting cells [55]. Thus, the possibility that IL-10 secreted by CD4^+^ T cells during infection is responsible of MHC class II decrease on DCs cannot be ruled out.

To evaluate the influence of glycoconjugates parasites in the specific stimulatory capacity of DCs conditioned with *F*. *hepatica* components, we co-cultured FhTE-pulsed BMDCs with CD4^+^ T cells from infected and non-infected animals. Cultures from infected animals produced high levels of IL-4, IL-5 and IL-10 with low levels of IFNγ. However, when parasite oxidized components were used for BMDC loading, CD4^+^ T cells from infected animals reduced their capacity to produce IL-4 and IL-10, indicating that parasite glycoconjugates are involved in the DC-triggered production of IL-4 and IL-10 by T cells. Thus, our work demonstrates for the first time the role of *F*. *hepatica* glycoconjugates in immunomodulating the host immune response during infection and, in particular, by modulating T-cell stimulatory capacity of DCs. Regulatory DCs can exert their functions through different mechanisms, such as by decreasing their capacity of antigen presentation or by inducing regulatory T cells able to suppress inflammatory Th1/Th17 responses [56]. In this context, it is interesting to remark that antigen presenting cells from the peritoneal cavity of infected animals, but not CD11c^+^ spleen cells, have limited capacity to induce effector T cell response [4], although their ability to directly induce IL-10 secreting regulatory T cells has not been evaluated.

In an attempt to gain more insight in the process of glycan-mediated immune-modulation of DCs, we focused on the study of the effects of glycoconjugates on DC-maturation *in vitro*. Importantly, previous reports demonstrate the capacity of *F*. *hepatica* components to modulate *in vitro* TLR-induced maturation of DCs and/or their stimulatory function [5–7, 16]. However, the role of parasite glycans in this immune-modulation had not been previously addressed. When BMDCs were matured in the presence of parasite components they secreted higher levels of IL-10 and lower levels of IL-6 and IL-12/23p40 than cells stimulated only with LPS. On the other hand, when DCs were matured with LPS in presence of oxidized parasite components the production levels of IL-6, IL-10 and IL12/23p40 were restored, indicating that glycoconjugates from *F*. *hepatica* mediate modulation of TLR-induced maturation of DCs. One possible mechanism that can account for this immune-modulation is by triggering carbohydrate specific receptors, such as CLRs, that can cross-talk with TLR-stimulation. Indeed, we found that both binding and uptake of parasite components by DCs were inhibited with EDTA or with Man, suggesting a CLR mediated process of recognition and uptake of parasite glycoconjugates through Man-containing glycans. On the other hand, GalNAc inhibited binding but not uptake by DCs, indicating that GalNAc-residues on parasite components can interact with receptors on the surface of DCs, but do not mediate antigen internalization. Strikingly, incubation with laminarin, a ligand of Dectin-1, did not inhibit either the binding or the uptake of parasite components present on FhTE by BMDCs. It has been recently published that Dectin-1 is involved in the induction of CD4^+^ T cell anergy by macrophages [23]. However, it should be noted that in this case excretory-secretory products from *F*. *hepatica* were used. Since the immunomodulatory properties of these parasite products on macrophages were found to depend on Dectin-1 signaling, it is likely that the different carbohydrate composition and/or nature of molecules present on FhTE (total lysate) and excretory-secretory parasite products determine the different CLR-mediated signaling triggered by *F*. *hepatica* components. In this sense, in previous elegant studies using *Helicobacter pylori* variants which differ in their expression of Lewis glycan antigens, only the variants expressing the Lewis antigen were capable to bind to DC-SIGN and block the induction of a specific Th1 response, while Lewis-negative *H*. *pylori* variants were not [57]. Eventual studies on the elucidation of the carbohydrate moieties present on *F*. *hepatica* total lysate and excretory-secretory products will be decisive to explain their different immunomodulatory properties.

By carrying out DC-maturation assays in presence of Man, we provide evidence that a Man-specific CLR expressed on DC surface mediates the immunomodulatory effects of *F*. *hepatica* components. Indeed, Man incubation restored the production levels of IL-10 and IL-12/23p40, but not those of IL-6, MIP-1α or MIP-2. Several CLRs have been reported to cross-talk with TLR-signaling on DCs as well as on other myeloid cells, inducing an increase of IL-10 and a decrease of pro-inflammatory cytokines [29, 58]. Furthermore, glycans from helminths can interact with CLRs on DCs and regulate their maturation. For instance, *S*. *mansoni*, glycans are recognized by the Man receptor resulting in a Th2-polarized cell response [10]. Also, the glycosylated molecules of the whipworm *T*. *suis* interact with the Man receptor, DC-SIGN and MGL, which recognize Man and terminal GalNAc residues, respectively, and suppress TNFα production by DCs stimulated with LPS [15]. According to our experimental results obtained with different inhibitors of molecules that participate in Man-specific CLR signaling, the phosphatase SHP2 would participate in the Man-triggered signaling. This indicates a possible role of SHP2-dependent CLR signaling in the recognition of *F*. *hepatica* glycans and cross-talk with TLR4-tiggering. One possible candidate is DCIR, a CLR identified on mouse BMDCs [59] and other myeloid cells [29], that can modulate TLR-induced gene expression at the transcriptional or post-transcriptional level. However, DCIR does not induce gene expression in the absence of other PRR signaling [29]. DCIR-triggering induces the phosphorylation of its ITIM (Immunoreceptor Tyrosine-based Inhibitory Motif), which recruits the phosphatases SHP1 or SHP2 to its cytoplasmic domain [60–61], and results in the inhibition of TLR8-mediated IL-12 and TNFα production or TLR9-induced IFNα and TNFα production by DCs [62–63]. Experiments are on their way to determine the role of DCIR in the immune-modulation induced by *F*. *hepatica* components, by specifically silencing DCIR expression on BMDCs.

The possible role of DCIR in the recognition of Man residues from *F*. *hepatica* and in mediating the cross-talk with TLR4 signaling could also explain the fact that both IL-10^+^ DCs and IL-12^+^ DCs were identified in the spleen from infected animals. Indeed, splenic DC subsets differentially express membrane molecules, some of which are CLRs [64–65]. In mouse spleen two different DC-subsets can be identified, CD11b^+^ DCs and CD8α^+^ CD11b^-^ DCs, which differ in antigen presentation and T-cell stimulatory capacity [64]. In fact, CD8α^+^ splenic DCs are the major subset responsible for cross-presenting cellular antigens [66]. On the other hand, CD8α^−^ DCs preferentially present antigen to CD4^+^ T cells [67]. These DC subsets also express different sets of surface molecules, including distinct CLRs. Interestingly splenic CD8α^-^ DCs have been reported to express DCIR, while CD8α^+^ DCs do not [67]. Thus, taking these findings into account, one could speculate that CD8α^-^ DCs expressing DCIR are the main target of immune regulation between different DCs found in mouse spleen by *F*. *hepatica* Man-containing glycans. Furthermore, a possible DCIR signaling could explain the immunomodulatory effects of *F*. *hepatica* glycoconjugates on splenocytes, since splenic B cells also express DCIR [60]. Additional experiments are needed to support this hypothesis.

In spite of the hypothesized role of DCIR in mediating immune-modulation by *F*. *hepatica* Man-glycans, inhibition culture assays with Man did not restore IL-6, MIP-1α or MIP-2 production. Thus, it is likely that another receptor or glycan structure is participating in the immune-modulation process that could not be detected with our assays. It has recently been reported that *F*. *hepatica* glycans interact with the DC-SIGN receptor on human DCs. Interestingly when DC-SIGN interacts with Man-rich glycans, modulates the production of pro-inflammatory TLR-induced cytokines by a pathway that depends on the activation of Raf-1 [68]. In fact, the interaction of *F*. *hepatica* glycans and DC-SIGN on human DCs, in presence of LPS, induces follicular T helper cell differentiation via IL-27 [16]. However, although the authors assume that this process is Fuc-dependent, the identity of the immunomodulatory glycoconjugates from *F*. *hepatica* was not investigated. In mice, however, there are eight DC-SIGN homologs that do not recognize exactly the same glycans as DC-SIGN [69]. According to their glycan specificity, SIGNR1 and SIGNR3 are the closest candidates to fulfill DC-SIGN function in mice [69]. Interestingly, SIGNR3 has been shown to modulate *M*. *tuberculosis* immune responses, by a signaling dependent on the tyrosine kinase Syk [70]. Indeed, resistance to *M*. *tuberculosis* is impaired in SIGNR3-deficient animals [70]. The fact that our results demonstrate that modulation of TLR-induced maturation of DCs by *F*. *hepatica* components does not depend on either Raf-1- or Syk-mediated signaling would exclude the participation of these receptors in this process. Nevertheless, additional studies are needed to elucidate a possible role of other SIGNR receptors in the immune modulation by *F*. *hepatica* on mice DCs.

In conclusion, the results reported here demonstrate that glycoconjugates from *F*. *hepatica* are involved in the induction of high levels of IL-10 and IL-4 by the parasite and are in agreement with the increasing evidence supporting a role for helminth glycans in regulation of the host immune shift toward a Th2/regulatory response via induction of IL-10. Furthermore, we show that *F*. *hepatica* glycoconjugates interact with DCs and modulate DC-function and -maturation by a SHP2-dependent CLR that is inhibited by Man residues. We are currently working on the identification of these glycans and the C-type lectin receptors on DCs that participate in their recognition. These results contribute to the understanding of the role of parasite glycans in the modulation of the host immunity and might be useful in the design of vaccines against fasciolosis.

## Supporting Information

S1 Fig DCs from infected animals present a semi-mature phenotype. Mice (n = 5 per group) were orally infected with 5, 10 or 15 metacercariae in PBS (infected mice). PBS alone served as a control (non-infected mice). Three weeks later mice were bled, sacrificed h and spleens, PECs and livers were removed. Fluke burden was analyzed by counting parasites on livers and alanine transaminase activity was measured in sera (**A**). Cell suspensions from spleens (**B**) or PECs (**C**) were incubated with anti-CD11c, -MCHII, -CD40, -CD80 and–CD86 specific antibodies and analyzed by flow cytometry Results are expressed as the mean of three independent experiments (±SD, indicated by error bars). Asterisks indicate statistically significant differences (*p < 0.01) with respect to cells from non-infected animals.
(PDF)

S2 Fig Gate selection for the study of dendritic cells and macrophages in infected animals. BALB/c animals infected with 10 metacercariae were sacrificed at 3 wpi. Then splenocytes (A) or PECs (B) were stained with CD3-APC, F4/80-FITC, MHCII-PE and CD11c-PECy7 antibodies, followed by permeabilization and staining with IL-10 and IL-12 PerCP-conjugated specific antibodies. Dendritic cells were defined as CD3^-^ F4/80^-^ CD11c^hi^ cells, while macrophages were defined as CD3^-^ CD11c^-^ F4/80^+^ cells.
(PDF)

S3 Fig *F*. *hepatica* infection promotes the recruitment of IL-10^+^ macrophages both at the peritoneal cavity and spleen. Mice (n = 5 per group) were orally infected with 15 metacercariae in PBS (infected mice). PBS alone served as a control (non-infected mice). Mice were sacrificed one, two and three weeks after the infection and spleens and PECs were removed. Splenocytes (**A**) and PECs (**B**) were counted and the presence of F4/80^+^ CD11c^-^ cells was analyzed by flow cytometry by staining cells with specific antibodies. Splenocytes (**C**) and PECs (**D**) were also incubated with anti-MCHII, permeabilized, and intracellular stained with anti-IL-10 and IL-12/23p40 antibodies for 30 min at 4°C. Cells were analyzed on a flow cytometer. Results are expressed as the mean of three independent experiments (±SD, indicated by error bars). Asterisks indicate statistically significant differences (*p < 0.01) with respect to cells from non-infected animals.
(PDF)
