# Supplementary material for: Glycans from Fasciola hepatica Modulate the Host Immune Response and TLR-Induced Maturation of Dendritic Cells
Source: PLoS Negl Trop Dis. 2015 Dec 31;9(12):e0004234. doi: 10.1371/journal.pntd.0004234 (PMC4697847; doi:10.1371/journal.pntd.0004234)

S1 Fig.

A

		N° Mice with macroscopic liver damage	Fluke burden	Transaminase Activity (U/L)
	Non-infected	0	- - -	9 ± 2
Infected	5 mets	3	- - -	260 ± 32
Infected	10 mets	5	0,4 ± 0,2	324 ± 11
Infected	15 mets	5	1,8 ± 0,4	425 ± 47

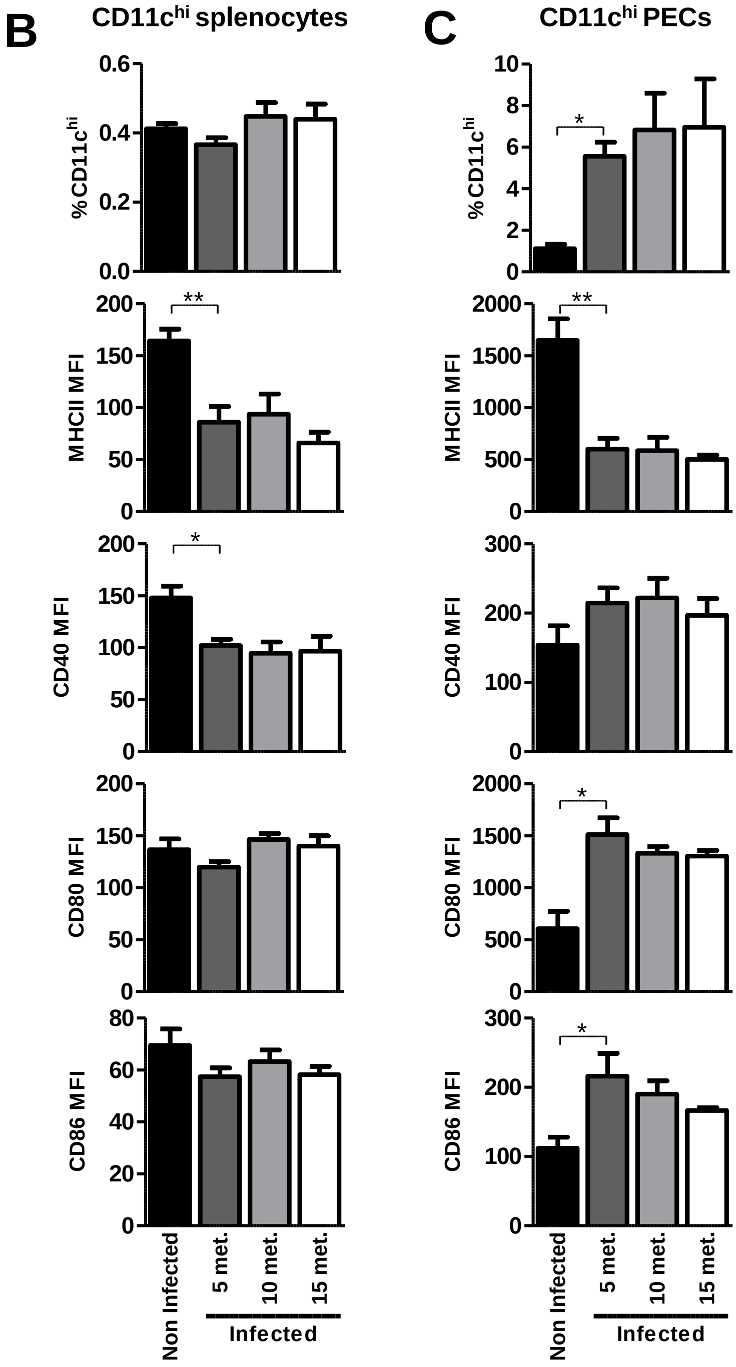

Supplement: S1 Fig — Mice (n = 5 per group) were orally infected with 5, 10 or 15 metacercariae in PBS (infected mice). PBS alone served as a control (non-infected mice). Three weeks later mice were bled, sacrificed h and spleens, PECs and livers were removed. Fluke burden was analyzed by counting parasites on livers and alanine transaminase activity was measured in sera (A). Cell suspensions from spleens (B) or PECs (C) were incubated with anti-CD11c, -MCHII, -CD40, -CD80 and–CD86 specific antibodies and analyzed by flow cytometry Results are expressed as the mean of three independent experiments (±SD, indicated by error bars). Asterisks indicate statistically significant differences (*p < 0.01) with respect to cells from non-infected animals. (PDF) [file pntd.0004234.s001.pdf]
