# Supplementary figures and images for: Glycans from Fasciola hepatica Modulate the Host Immune Response and TLR-Induced Maturation of Dendritic Cells
Source: PLoS Negl Trop Dis. 2015 Dec 31;9(12):e0004234. doi: 10.1371/journal.pntd.0004234 (PMC4697847; doi:10.1371/journal.pntd.0004234)

S2 Fig.

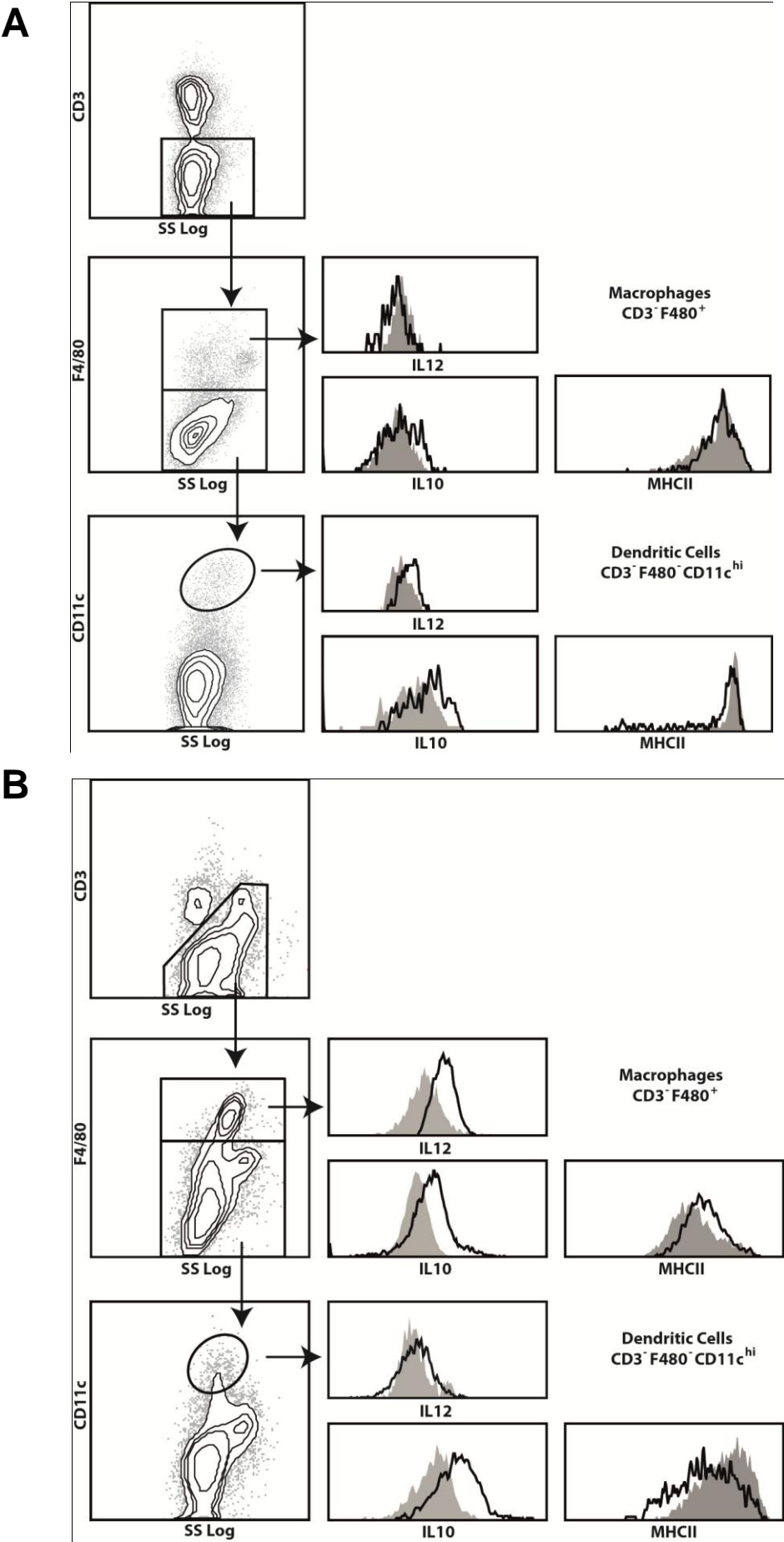

Supplement: S2 Fig — BALB/c animals infected with 10 metacercariae were sacrificed at 3 wpi. Then splenocytes (A) or PECs (B) were stained with CD3-APC, F4/80-FITC, MHCII-PE and CD11c-PECy7 antibodies, followed by permeabilization and staining with IL-10 and IL-12 PerCP-conjugated specific antibodies. Dendritic cells were defined as CD3- F4/80- CD11chi cells, while macrophages were defined as CD3- CD11c- F4/80+ cells. (PDF) [file pntd.0004234.s002.pdf]

# S3 Fig.

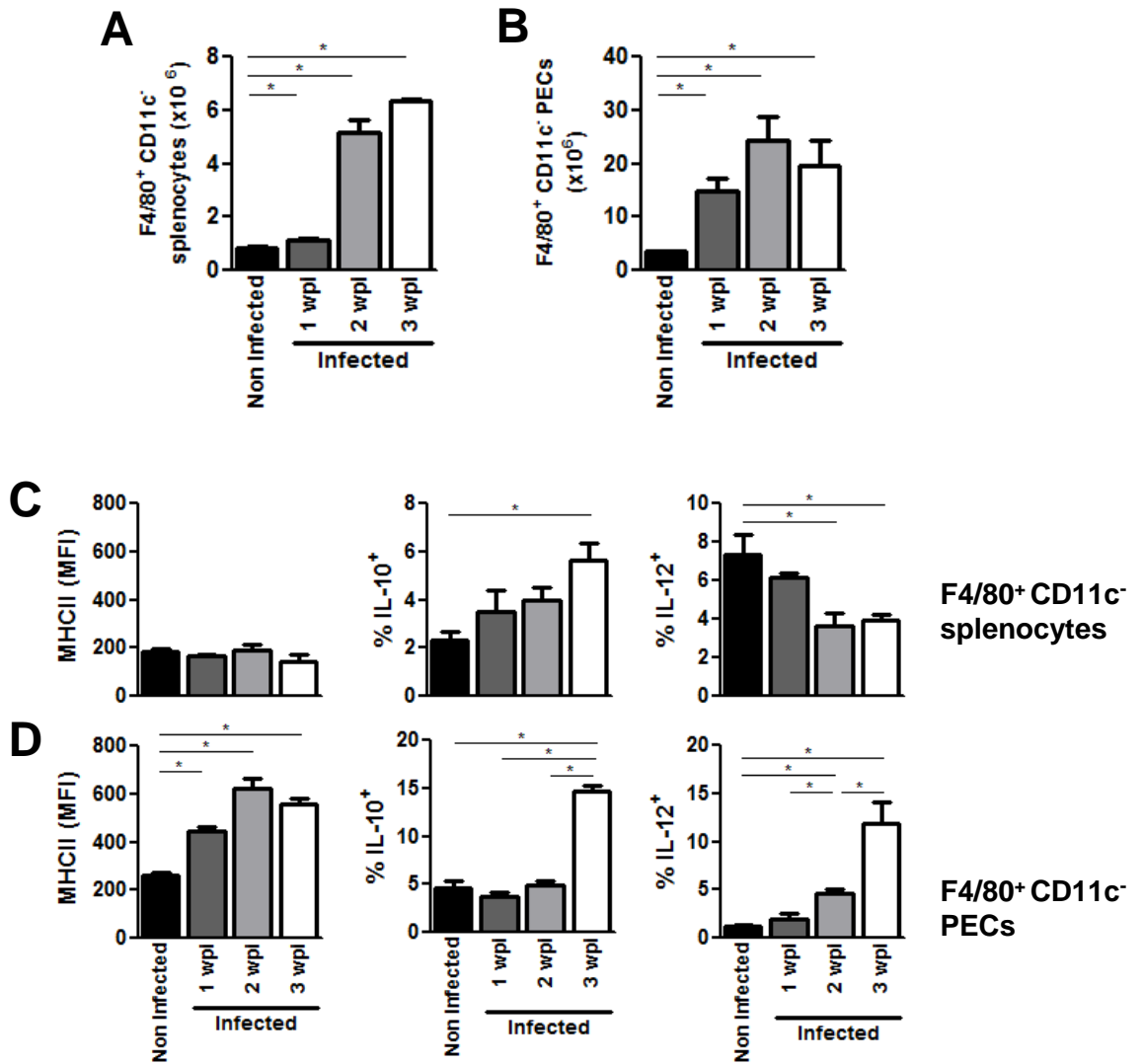

Supplement: S3 Fig — Mice (n = 5 per group) were orally infected with 15 metacercariae in PBS (infected mice). PBS alone served as a control (non-infected mice). Mice were sacrificed one, two and three weeks after the infection and spleens and PECs were removed. Splenocytes (A) and PECs (B) were counted and the presence of F4/80+ CD11c- cells was analyzed by flow cytometry by staining cells with specific antibodies. Splenocytes (C) and PECs (D) were also incubated with anti-MCHII, permeabilized, and intracellular stained with anti-IL-10 and IL-12/23p40 antibodies for 30 min at 4°C. Cells were analyzed on a flow cytometer. Results are expressed as the mean of three independent experiments (±SD, indicated by error bars). Asterisks indicate statistically significant differences (*p < 0.01) with respect to cells from non-infected animals. (PDF) [file pntd.0004234.s003.pdf]
